# Introducing Photochemical Action Plots as a Tool for Unlocking On–Off Switchable Behavior in a Polymeric Eosin Y Photocatalyst

**DOI:** 10.1002/anie.202502890

**Published:** 2025-05-12

**Authors:** Sebastian Gillhuber, Alicia K. Finch, Joshua O. Holloway, Hendrik Frisch, Florian Weigend, Christopher Barner‐Kowollik, Peter W. Roesky

**Affiliations:** ^1^ Institute of Inorganic Chemistry Karlsruhe Institute of Technology (KIT) Engesserstraße 15 76131 Karlsruhe Germany; ^2^ School of Chemistry and Physics Queensland University of Technology (QUT) 2 George Street Brisbane QLD 4000 Australia; ^3^ Centre for Materials Science Queensland University of Technology (QUT) 2 George Street Brisbane QLD 4000 Australia; ^4^ Institute for Quantum Materials and Technologies Karlsruhe Institute of Technology (KIT) Hermann‐von‐Helmholtz‐Platz 1 76344 Eggenstein‐Leopoldshafen Germany; ^5^ Institute of Nanotechnology (INT) Karlsruhe Institute of Technology (KIT) Hermann‐von‐Helmholtz‐Platz 1 76344 Eggenstein‐Leopoldshafen Germany; ^6^ Institute of Functional Interfaces (IFG) Karlsruhe Institute of Technology (KIT) Hermann‐von‐Helmholtz‐Platz 1 76344 Eggenstein‐Leopoldshafen Germany

**Keywords:** Action plots, Eosin Y, Photocatalysis, Single‐chain nanoparticles, Switchable catalysis

## Abstract

Photochemical action plots are a powerful tool for investigating the wavelength‐dependent efficiency of photochemical processes. Herein, we apply the photochemical action plot methodology developed by the Barner–Kowollik team to a photocatalytic reaction, resulting in the first examples of photocatalytic action plots, paving the way for future in‐depth explorations of the wavelength dependence of similar reactions. Specifically, we investigate the wavelength dependence of the catalytic oxidation capabilities of an Eosin Y functionalized polymer photocatalyst (**P1**) as well as a small molecule representative of the polymer bound Eosin Y moieties (**EY**) for the oxidation of triphenylphosphine. Introduction of zinc(II) ions into the system proved to drastically influence the optical absorption properties of both catalysts, accompanied by a pronounced influence on their wavelength‐dependent reactivity profiles which is not predictable based on the absorption spectra. For **P1**, these changes can be reversed by metal‐mediated single‐chain nanoparticle (SCNP) formation upon base addition, giving access to a stimuli‐responsive polymeric photocatalyst. Detailed analysis of the photocatalytic action plots enabled the identification of a suitable wavelength for the realization of an on‐off switchable polymeric photocatalytic system.

## Graphical abstract

The photochemical action plot methodology developed by the Barner–Kowollik team provides a powerful tool for the investigation of the wavelength dependence of light‐driven reactions. Herein, the methodology is applied to a photocatalytic reaction, resulting in the first examples of photocatalytic action plots. The insights gained from the photocatalytic action plot analysis enable on‐off switching of the activity of a polymeric photocatalyst.

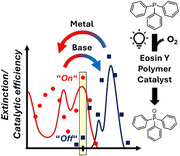

## Introduction

Photochemistry is at the heart of life on earth, driving key processes such as natural photosynthesis or helping to harvest sunlight to satisfy our energy demands to enable pathways toward a more sustainable future.^[^ ^1^ ^]^ The research field of photochemistry focuses on the exploration of chemical reactions driven by light irradiation.^[^ ^2^, ^3^ ^]^ In the last decades, tremendous progress has been made toward, for instance, unprecedented synthetic methodologies,^[^ ^4^, ^5^ ^]^ the manufacturing of new materials,^[^ ^6^, ^7^ ^]^ medical applications,^[^ ^8^, ^9^ ^]^ or photocatalytic transformations.^[^ ^10^, ^11^, ^12^, ^13^ ^]^ Traditionally, non‐monochromatic light sources, such as mercury, deuterium, or fluorescent lamps, have been employed to drive photochemical processes, and routinely the optical absorption spectrum of a reaction mixture has been utilized as a guide to choose a suitable irradiation wavelength.^[^ ^14^ ^]^ However, a body of work on the careful wavelength‐by‐wavelength mapping of photochemical reactions by the Barner–Kowollik team over the last decade, and recently others, has demonstrated the oftentimes poor correlation between the optical absorption properties of a molecule and its photochemical reactivity.^[^ ^14^, ^15^, ^16^, ^17^, ^18^, ^19^, ^20^, ^21^ ^]^ The wavelength dependence of a photochemical reaction can conveniently be monitored via the well‐established photochemical action plot methodology.^[^ ^14^, ^15^, ^16^, ^22^ ^]^ Specifically, a reaction mixture is subjected to irradiation with an identical and defined number of photons from a monochromatic light source at different wavelengths and the efficiency of the reaction at each wavelength probed by a suitable analytical technique. So far, efforts have mainly been focused on the exploration of bond breaking or forming reactions and the efficiency of photoinitiators for polymerizations.^[^ ^15^, ^23^ ^]^

In our ongoing research interest in different photochemical systems, we recently observed beneficial effects of anchoring dyes onto polymeric support for homogeneous photocatalytic oxidation reactions. In detail, Rose Bengal‐functionalized intramolecularly folded macromolecules, so‐called single‐chain nanoparticles (SCNPs), showed an up to three‐times increased turnover number for the oxidation of nonpolar alkenes compared to free Rose Bengal in solution.^[^ ^24^ ^]^

Intrigued by this finding, our interest moved to the wavelength dependence of the photocatalytic activity of dyes in related SCNP‐like environments. In recent years, especially the xanthene dye Eosin Y has found manifold applications in photocatalysis due to its broad application scope combined with low‐cost commercial availability.^[^ ^25^, ^26^ ^]^ Visible light irradiation of Eosin Y leads to the initial population of an excited singlet state, which rapidly undergoes conversion to the lowest energy excited triplet state with a high intersystem crossing quantum yield.^[^ ^27^, ^28^ ^]^ The population of the triplet state manifold makes Eosin Y prone to triplet energy transfer, enabling, for instance, the efficient generation of singlet oxygen. Continued irradiation of catalytic amounts of Eosin Y in the concomitant presence of triplet oxygen and suitable oxidizable substrates enable photocatalyzed oxidation reactions with the in situ generated singlet oxygen.

In the current contribution we aim at critically expanding the versatility of the photochemical action plot methodology by translating the concept to photocatalytic reactions, resulting in the first examples of photocatalytic action plots. To achieve this, we investigate the wavelength dependence of the catalytic activity of a derivative of the well‐known xanthene dye Eosin Y.^[^ ^25^, ^26^ ^]^ We compare the wavelength‐dependent catalytic activity of polymer‐bound Eosin Y functionalities to that of an analogue small molecule Eosin Y derivative and demonstrate how the insights gained from the photocatalytic action plot analysis can be employed to realize an on‐off switchable polymeric photocatalyst.

## Results and Discussion

### Photocatalytic Action Plots of P1 and EY

**Figure 1:**
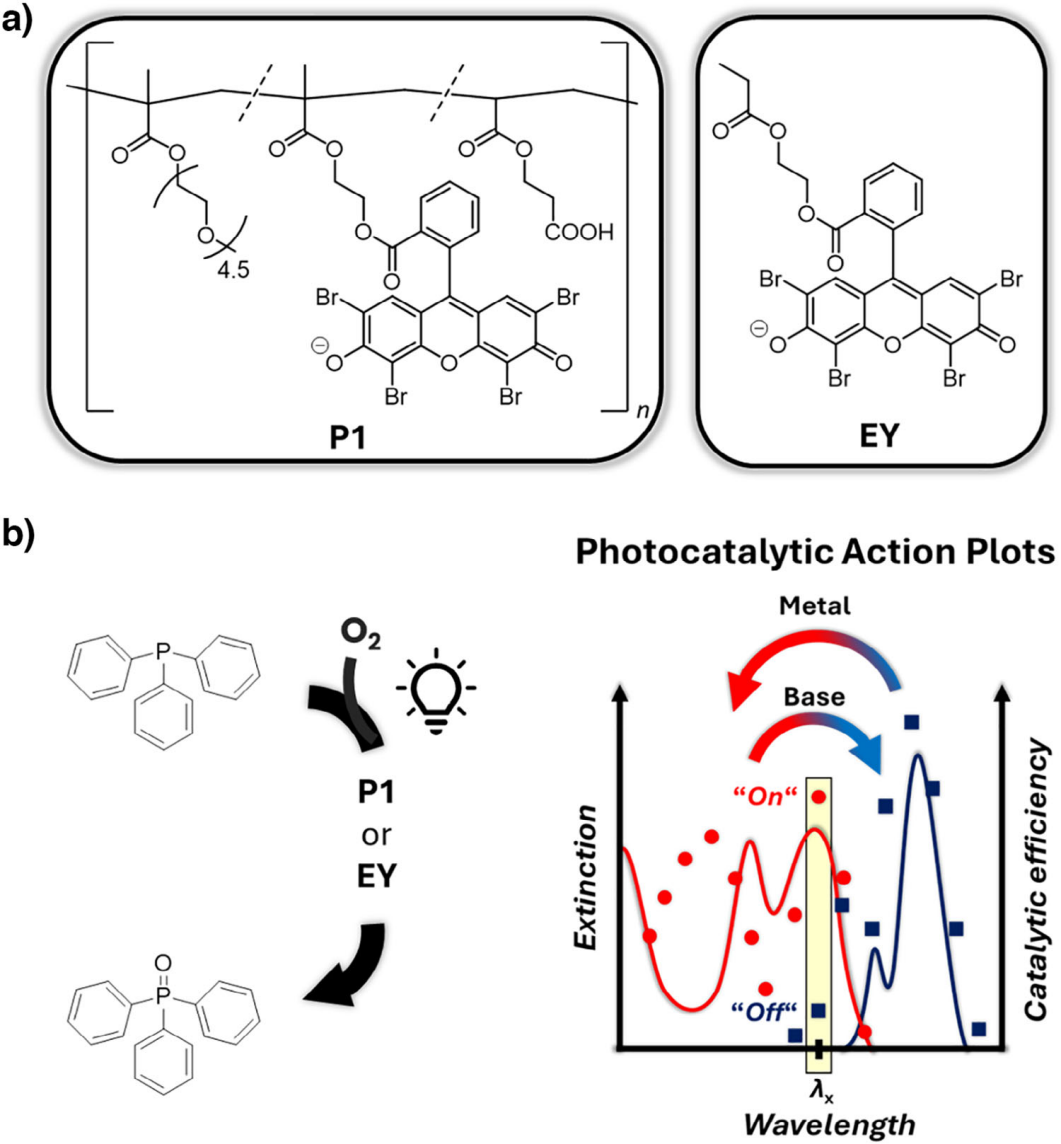
Overview. a) Chemical structures of the Eosin Y copolymer poly(PEGMEMA‐*co*‐EYMA‐*co*‐2CEA) (Polymer **P1**) and the small molecule Eosin Y derivative **EY**. b) Schematic overview of the work described herein. The irradiation wavelength dependence of the **P1**‐ or **EY**‐photocatalyzed ambient oxygen mediated oxidation of triphenylphosphine is investigated using the photochemical action plot methodology. Metal and base addition to the reaction mixture act as chemical stimuli influencing the optical absorption spectrum and the wavelength‐dependent photocatalytic activity, enabling on‐off switching of the catalytic activity at a particular wavelength *λ*_x_ identified from the photocatalytic action plots.

Initially, we prepared a copolymer of poly(ethylene glycol) methyl ether methacrylate (PEGMEMA, average *M*_n_ = 300 g·mol^−1^), 2‐carboxyethyl acrylate (2CEA) and the Eosin Y methacrylate monomer 2‐(methacryloyloxy)ethyl 2‐(2,4,5,7‐tetrabromo‐6‐hydroxy‐3‐oxo‐3*H*‐xanthen‐9‐yl)benzoate (EYMA) by reversible addition‐fragmentation chain transfer (RAFT)^[^ ^29^, ^30^ ^]^ polymerization (Polymer **P1**, Figure 1a). The PEGMEMA backbone provides the polymer with so *λ* lubility in a wide range of solvents, while the 2CEA moieties enable efficient post‐polymerization modification reactions, relevant to the SCNP formation process described below. The copolymer composition was analyzed by ^1^H nuclear magnetic resonance (NMR) spectroscopy. The successful copolymerization process was evident from the presence of the expected characteristic resonances of the PEGMEMA, 2CEA, and EYMA moieties, respectively (Figure S9 for detailed resonance assignments). Size‐exclusion chromatography (SEC) measurements of **P1** in *N,N*‐dimethylacetamide (DMAc) gave an indication of the number‐averaged molar mass of *M*_n_ = 43900 g·mol^−1^ and dispersity of *Ð* = 1.5. The successful inclusion of the Eosin Y functionalities into **P1** was additionally verified by ultraviolet‐visible (UV–vis) absorption spectroscopy measurements in acetonitrile, showing an intense extinction maximum at *λ* = 540 nm and a less intense peak at *λ* = 504 nm (Figure S15), characteristic of monoanionic Eosin Y functionalities.^[^ ^31^ ^]^ It is worth noting that the complexity stemming from tautomerism and delicate acid–base equilibria usually complicating the photochemistry of Eosin Y derivatives is reduced in the systems considered in the current study due to the esterification of the carboxylic acid functionality,^[^ ^11^, ^32^ ^]^ preventing lactonization and proton transfer at this position. Thus, proton transfer equilibria are limited to the hydroxyl functionality on the xanthene core.

As outlined in detail above, irradiation of Eosin Y derivatives in the presence of ambient oxygen leads to the generation of singlet oxygen which can be employed for catalytic in situ oxidation reactions. In the context of our ongoing research on the careful wavelength‐resolved investigation of photochemical processes, we were particularly interested in the wavelength dependence of the catalytic oxidation capability of the polymer bound Eosin Y functionalities within **P1**. Commonly, the photochemical action plot method is applied to primary photochemical reactions triggered directly by light.^[^ ^14^, ^15^, ^16^ ^]^ Its application to systems involving secondary, non‐photochemical reactions initiated by the products of primary photochemical processes is currently limited to photoinitiated polymerizations,^[^ ^17^, ^23^ ^]^ and catalytic transformations have not yet been investigated. We note, however, that wavelength‐resolved reactivity studies of photocatalytic reactions in general have been conducted before. In particular, action spectra are a well‐established tool in the semiconductor photocatalysis field.^[^ ^33^, ^34^, ^35^ ^]^

**Figure 2:**
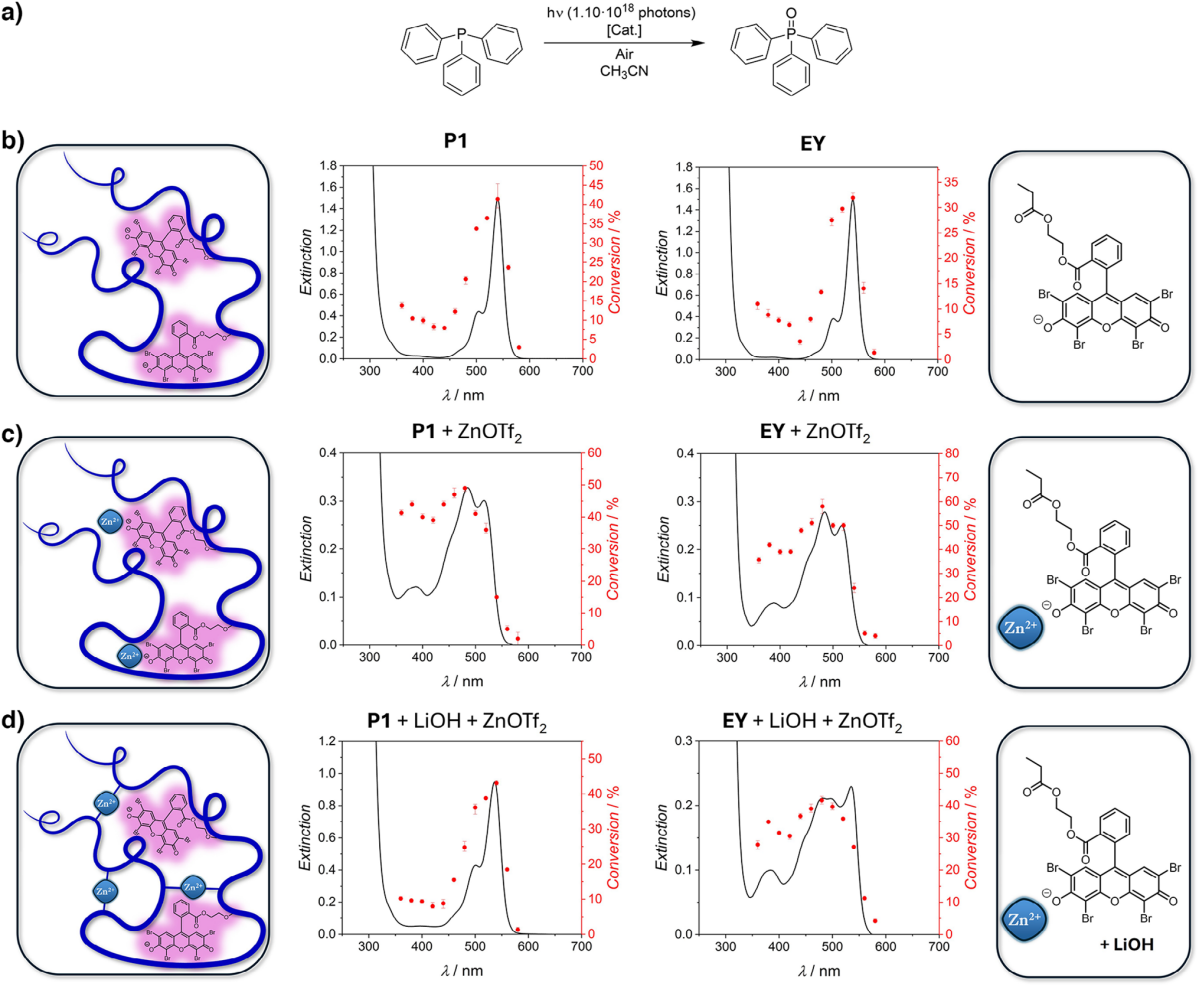
Photocatalytic action plots. a) Reaction equation of the photosensitized oxidation of triphenylphosphine in acetonitrile under ambient conditions. b) [Cat.]: **P1** (left) or **EY** (right) at a dye loading of 0.5 mol% with respect to triphenylphosphine. c) [Cat.]: **P1** (left) or **EY** (right) at a dye loading of 0.5 mol% in the presence of 7.5 mol% zinc(II) triflate (loadings with respect to triphenylphosphine). d) [Cat.]: **P1** (left) or **EY** (right) at a dye loading of 0.5 mol% in the presence of 30 mol% lithium hydroxide and 7.5 mol% zinc(II) triflate (loadings with respect to triphenylphosphine). In each case, black solid lines correspond to extinction spectra of the respective reaction mixtures. Red dots refer to average conversion of triphenylphosphine to triphenylphosphine oxide upon irradiation with 1.10 · 10^18^ photons within 5 min at each wavelength. Error bars indicate the lowest and highest determined conversions at each wavelength, respectively.

To probe, for the first time, the irradiation wavelength dependence of a photocatalytic process using our photochemical action plot methodology, we employed **P1** as a photocatalyst for the oxidation of triphenylphosphine to triphenylphosphine oxide^[^ ^36^ ^]^ as a model reaction, which can conveniently be followed by ^1^H NMR spectroscopy (Figures 1b and 2a). In detail, we irradiated an acetonitrile solution of **P1** with a dye concentration of approximately 13.4 µmol·L^−1^ (refer to Supporting Information Chapter 3.4 for details) in the presence of circa 200 equiv. of triphenylphosphine (with respect to the dye) with 1.10 · 10^18^ photons at each wavelength between 360 and 580 nm in 20 nm increments and quantified the conversion of triphenylphosphine to triphenylphosphine oxide by ^1^H NMR spectroscopy. Superimposition of the resulting conversions in dependence on the irradiation wavelength with the UV–vis spectrum of the reaction mixture results in the photocatalytic action plot depicted in Figure 2b. Each data point was recorded in triplicate. The data points refer to mean conversions, and the error bars indicate the lowest and highest determined conversions at each wavelength, respectively. It is evident that the highest conversion from the starting material to the catalysis product was achieved upon irradiation at 540 nm, coinciding with the extinction maximum in the UV–vis spectrum of the reaction mixture. Further, the wavelength‐dependent reactivity pattern resembles the absorption profile with a pronounced decrease in absorptivity and reactivity around the respective peak values.

To probe the influence of the polymeric environment on the wavelength dependence of the photocatalytic efficiency, we additionally conducted the photocatalytic action plot experiments with the small molecule Eosin Y derivative 2‐(propionyloxy)ethyl 2‐(2,4,5,7‐tetrabromo‐6‐hydroxy‐3‐oxo‐3*H*‐xanthen‐9‐yl)benzoate (**EY**, Figure 1a), representative of the Eosin Y functionalities within **P1** under otherwise identical conditions. The resulting photocatalytic action plot (Figure 2b) is qualitatively identical to that obtained employing **P1** as the catalyst, with the highest catalytic efficiency at 540 nm and a pronounced drop around the extinction maximum. It is noted, however, that the obtained average conversions are consistently higher over the entire wavelength interval probed when **P1**, instead of **EY**, is employed as the catalyst, hinting at a beneficial effect of the polymeric environment and in agreement with our previous observations.^[^ ^24^ ^]^ The effect is most pronounced between 480 and 560 nm with up to 10% higher average conversions when employing the polymeric photocatalyst.

### Effects of Metal Functionalization

As outlined above, the photochemical properties of Eosin Y and its derivatives are strongly dependent on delicate equilibria involving the hydroxyl and carboxyl functionalities. While the carboxyl group is unavailable for further modifications in the present case, the hydroxyl functionality is accessible to chemical stimuli, for instance, interactions with metal cations.^[^ ^37^ ^]^ Indeed, addition of the metal salt zinc(II) triflate to acetonitrile solutions of **P1** or **EY**, respectively, was found to exert a pronounced effect on the optical absorption spectra of the compounds. In detail, the extinction maximum at *λ* = 540 nm with the shoulder at *λ* = 504 nm disappeared upon addition of the metal source, accompanied by the emergence of several overlapping new bands with three distinct local extinction maxima centered at *λ* = 386, 485, and 516 nm, respectively (Figure 2b,c).

To obtain a more detailed understanding of the observed influence of metal addition on the electronic excitation spectra, time‐dependent density functional theory (TDDFT) calculations at the BLYP/def2‐TZVPP level of theory were performed (refer to Supporting Information Chapter 5 for computational details). The model compounds **EY‐Na**, representative of deprotonated **EY** as well as the Eosin Y moieties within **P1**, and **EY‐Zn**, representative of zinc(II) functionalized **EY** or **P1**, respectively, were considered (Figure S17a). Specifically, singlet vertical excitation energies and oscillator strengths of the 20 lowest energy excitations were calculated (Tables S11 and S12). Based on the determined values, electronic excitation spectra were simulated (Figure S17b). The shape of the simulated absorption spectrum of **EY‐Na** is in excellent agreement with the experimental UV–vis spectra of the reaction solutions employed for recording the photochemical action plots depicted in Figure 2b. According to the calculations, the highest intensity absorption peak is associated with two transitions (*i*_EY‐Na_ and *ii*_EY‐Na_) of similar excitation energies and oscillator strengths. The non‐relaxed difference density plots (Figure S17c) of the respective transitions are qualitatively identical and indicate a transfer of electron density from the xanthene unit to the attached substituted phenyl ring upon excitation. The shoulder toward higher excitation energies corresponds to a single excitation (*iii*_EY‐Na_) centered on the xanthene core. The pronounced influence of zinc functionalization on the optical absorption spectrum outlined above is also reflected in the calculations. Reminiscent of the experimental spectra of the reaction solutions employed for recording the photochemical action plots depicted in Figure 2c, the simulated electronic excitation spectrum of **EY‐Zn** features several overlaying absorption bands with three distinct local maxima (Figure S17b). Analogous to **EY‐Na**, the lowest energy excitation peak is constituted of two almost degenerate excitations (*i*_EY‐Zn_ and *ii*_EY‐Zn_) with similar oscillator strengths. The non‐relaxed difference density plots (Figure S17c) indicate a large contribution of the zinc ion to the respective transitions, with electron density being transferred from the Eosin Y moiety to the metal upon excitation. The highest intensity peak in the simulated spectrum is dominated by a single excitation (*iii*_EY‐Zn_) centered on the xanthene moiety. The highest energy absorption band features contributions of several energetically close lying transitions (*iv*_EY‐Zn_) mainly corresponding to a transfer of electron density from the xanthene core to the phenyl ring substituent.

To investigate the effects associated with the discussed changes in the optical properties on the catalytic transformation, the photocatalytic action plots of the Eosin Y‐sensitized triphenylphosphine oxidation employing the small molecule **EY** as well as polymer **P1**, each after the addition of zinc(II) triflate, as catalysts were studied (Figure 2c). Concentrations and irradiation conditions were kept identical to the previously discussed action plots (refer to Supporting Information Chapter 3.4 for experimental details). The pronounced changes in the optical absorption spectra of the catalysts upon metal functionalization are also reflected in the wavelength‐dependent reactivity profiles, with pronounced changes in the achievable conversion of specific wavelength regimes, which cannot be anticipated by inspection of the absorption spectra.^[^ ^16^ ^]^ Although in the absence of zinc(II) the highest conversion of triphenylphosphine to triphenylphosphine oxide was observed upon irradiation at 540 nm, the reactivity at this wavelength was drastically reduced after metal addition. Instead, the maximum catalyst efficiency was observed at 480 nm, with average conversions of 58% (**EY** + ZnOTf_2_) and 49% (**P1** + ZnOTf_2_), respectively. Overall, the achieved conversions were significantly higher in the wavelength range from 360 to 520 nm compared to the situation in the absence of the metal ions. Interestingly, the small molecule and polymeric catalysts performed similarly in terms of the achieved conversions upon irradiation at wavelengths between 360–460 nm and 560 –580 nm, respectively. However, in the range between 480 and 540 nm the small molecule catalyst showed a superior performance, with the difference to the polymeric catalyst being most pronounced at 520 nm, where the small molecule achieved a 14% increased average yield of the catalysis product compared to the polymer catalyst.

### Effects of Base Addition

As discussed above, the distinct influence of zinc(II) addition on the optical properties and catalytic performance of the Eosin Y‐based catalysts is expected to result from the direct interaction of the metal ions with the deprotonated Eosin Y hydroxy group. These interactions can be interrupted by the introduction of an additional ligand displacing the Eosin Y moieties from the inner zinc coordination sphere. Upon deprotonation, the carboxylic acid functionalities present in **P1** can exert this effect, thus enabling access to a stimuli responsive polymeric Eosin Y photocatalyst. Addition of the base lithium hydroxide to an acetonitrile solution containing **P1** and zinc(II) triflate reverted the influence metal addition showed on the optical absorption spectrum of **P1**, except for slightly decreased extinction values, indicating the successful recovery of the non‐complexed Eosin Y functionalities due to the complexation of the zinc(II) ions by the polymeric carboxylate groups (Figure 2b–d).

In contrast, the addition of lithium hydroxide to an acetonitrile solution of **EY** and zinc(II) triflate did not result in the recovery of the pristine **EY** extinction spectrum, highlighting that the presence of the carboxylate functionalities is critical to the effect observed for **P1** (Figure 2b–d). However, slight changes of the extinction profile with a partial recovery of the peak at *λ* = 540 nm were also observed for **EY**, indicating an interaction of the Zn(II)‐**EY** complex with the added hydroxide ions, presumably partially releasing **EY** from the inner zinc coordination sphere. The discrepancies in the effects base addition showed on the optical absorption spectra of **EY** and **P1** in the presence of zinc(II) triflate are also reflected in the wavelength‐resolved reactivity patterns of the photocatalytic triphenylphosphine oxidation as is evident from the photocatalytic action plots depicted in Figure 2d. In the case of **P1**, within the methodological error boundaries, the same wavelength‐dependent conversions of the catalysis substrate to the desired product were obtained as were for the pristine polymer (Figure 2b left), demonstrating the effective reversion of the metal functionalization of the Eosin Y moieties upon base addition. In the case of **EY**, on the other hand, the wavelength‐dependent reactivity pattern is not reminiscent of that of the pristine small molecule **EY** (Figure 2b right) and resembles the reactivity profile observed for **EY** in the presence of zinc(II) triflate instead (Figure 2c). Compared to **EY** in the presence of zinc(II) triflate only, base addition consistently reduced the substrate conversions by about 10% in the wavelength range from 360 to 520 nm, while the conversions remained almost uninfluenced in the region 540 to 580 nm.

To gather a more detailed understanding of the processes occurring upon metal functionalization of **EY** and **P1** as well as base addition, further experimental studies were undertaken. Figure S4 shows the aromatic region of the ^1^H NMR spectra of **EY** acquired in CD_3_CN in the absence and presence of zinc(II) triflate as well as in the concomitant presence of zinc(II) triflate and lithium hydroxide. It is evident that the addition of zinc(II) triflate to **EY** is accompanied by a downfield shift of the aromatic resonances, indicating removal of electron density from the xanthene core, in line with the expected interactions of the Lewis acidic metal ions with the dye molecules. This remains virtually unaltered upon lithium hydroxide addition, demonstrating that **EY** coordination to the zinc(II) ions is not disrupted by base addition.

When zinc(II) triflate is added to an acetonitrile solution of **P1**, dynamic light scattering (DLS) measurements (Figure S5) reveal an increase in the solvodynamic diameter from *D*_s_ = 7.3 nm (**P1**) to *D*_s_ = 11.5 nm (**P1** + ZnOTf_2_), showing that the metal ions are incorporated into the macromolecules, causing their swelling. Deprotonation of the polymer‐bound carboxylic acid functionalities proved to compact the structures again, resulting in a solvodynamic diameter of *D*_s_ = 8.6 nm (**P1** + ZnOTf_2_ + LiOH), suggesting the formation of SCNPs folded by the coordination of polymer carboxylate groups to the zinc ions. We note that the synthesis of dye‐functionalized or photocatalytically active SCNPs is a vital research area with a number of recent reports in the literature.^[^ ^24^, ^38^, ^39^, ^40^, ^41^, ^42^, ^43^, ^44^, ^45^, ^46^, ^47^ ^]^

### On‐Off Switchable Catalysis

The response of **P1** to chemical stimuli, altering the optical absorption properties and concomitantly the rate of the photocatalytic transformation of triphenylphosphine to triphenylphosphine oxide, serves as a blueprint for the realization of a chemically on‐off switchable polymeric photocatalyst system. Inspection of the photocatalytic action plots of the triphenylphosphine oxidation employing **P1** as the catalyst in the absence (Figure 2b) and presence (Figure 2c) of zinc(II) triflate, respectively, shows that the difference in catalytic activity is most pronounced at 440 nm.

**Figure 3:**
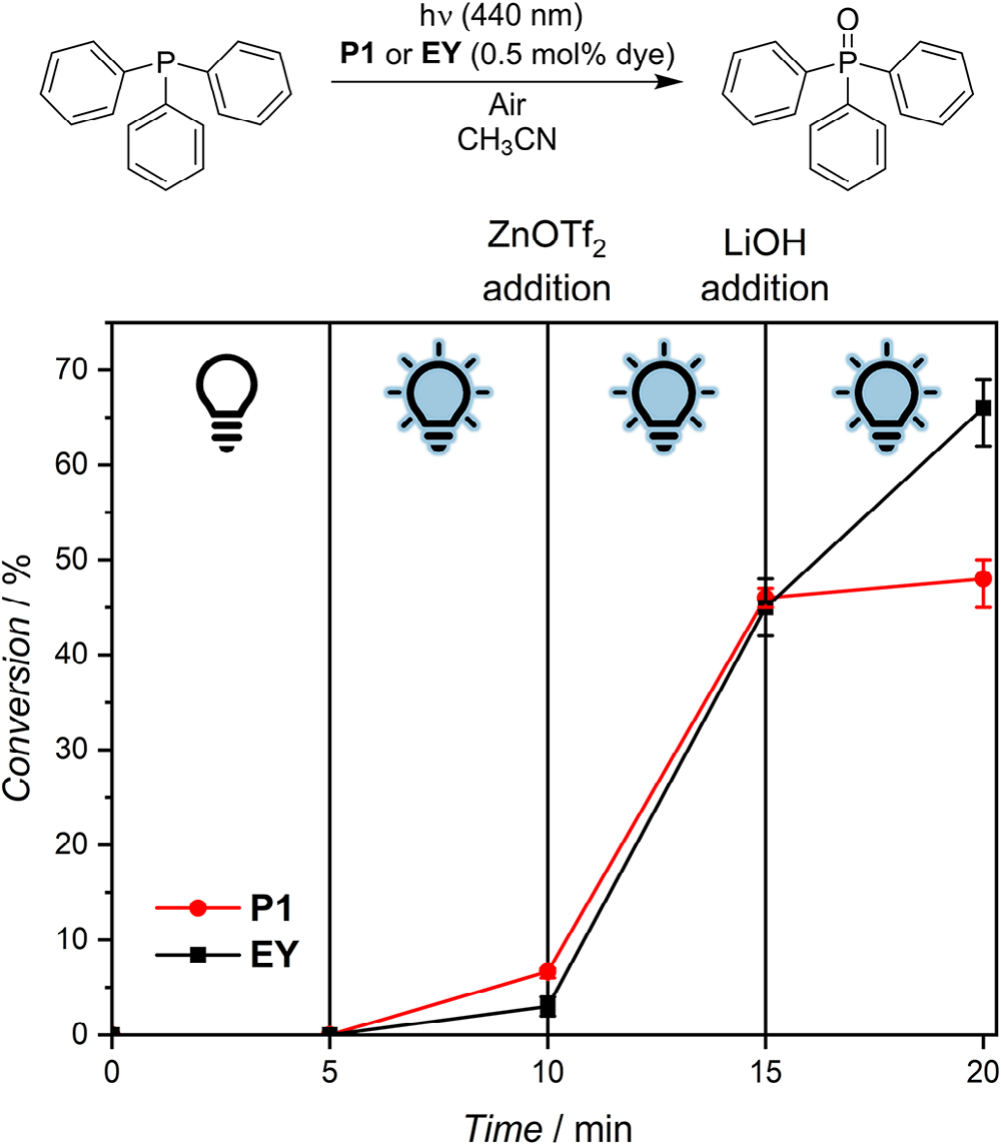
On‐off switchable catalysis. Top: Reaction equation. Bottom: Conversion of triphenylphosphine to triphenylphosphine oxide in the photosensitized oxidation catalyzed by **P1** (red) or **EY** (black) in acetonitrile under ambient conditions at a dye loading of 0.5 mol% with respect to the catalysis substrate. Without irradiation (0–5 min), upon irradiation at 440 nm (5–10 min, 1.10 · 10^18^ photons), and continued irradiation at 440 nm after subsequent addition of zinc(II) triflate (10–15 min, 1.10 · 10^18^ photons) and lithium hydroxide (15–20 min, 1.10 · 10^18^ photons). Data points show average conversions, and error bars indicate the lowest and highest determined conversions at each wavelength, respectively.

This insight gained from the photocatalytic action plot analysis makes 440 nm the most suitable wavelength to employ **P1** as an on‐off switchable photocatalyst. To demonstrate the applicability of this concept, **P1** was again employed as a catalyst for the photosensitized triphenylphosphine oxidation in acetonitrile (Figure 3). Without irradiation, no conversion of the starting material was observed, demonstrating that light is critical for the catalytic reaction to take place. After 5 min in the dark, the sample was irradiated with 1.10 · 10^18^ photons within 5 min at 440 nm. As expected, due to the local minimum of the catalytic efficiency in the corresponding photocatalytic action plot (Figure 2b) at this wavelength, only a low conversion of the substrate to the catalysis product of about 5% was reached, thus marking an inactive “*off*” state of the catalyst. The catalyst could be switched “*on*” by the addition of zinc(II) triflate, leading to a substantial increase of the catalytic rate upon 440 nm light exposure. Irradiation at 440 nm with 1.10 · 10^18^ photons over 5 min led to a substrate conversion of almost 50%. The catalyst could be switched “*off*” again by SCNP formation upon lithium hydroxide addition, causing displacement of the dye moieties from the inner zinc(II) coordination sphere by the polymer‐bound carboxylate functionalities, thus diminishing the effect of the metal ions on the catalytic rate. Indeed, continuing the irradiation at 440 nm with additional 1.10 · 10^18^ photons over 5 min only led to a negligible increase of the catalysis product yield, demonstrating the successful on‐off switching of the catalytic activity of the Eosin Y‐functionalized polymeric photocatalyst **P1**. Critically, the catalytic activity of the polymeric catalyst **P1** could be switched “*on*” again by the introduction of additional zinc(II) triflate to the system (Figure S8). As indicated by the photocatalytic action plot data discussed in detail above, the same could not be achieved with the small molecule catalyst **EY** under otherwise identical conditions (Figure 3). While addition of the metal salt successfully turned the catalytic activity “*on*”, it could not be switched “*off*” again by base addition as the hydroxide ions are not sufficiently strong ligands to quantitatively displace **EY** from the inner coordination sphere of the zinc ions, highlighting the opportunities provided by multifunctional polymer catalysts. Besides prospective applications in on‐demand switchable catalysis, interesting for instance for one‐pot cascade reactions in which a defined amount of a catalysis product is required with temporal control, our findings might also find practical use in other areas, e.g. in sensors for metal ions or selective wavelength‐orthogonal one‐pot parallel photoreactions.

## Conclusion

We critically expand the versatility of the well‐established photochemical action plot methodology by translating the concept to photocatalytic reactions, resulting in the first examples of photocatalytic action plots following the methodology developed by the Barner–Kowollik team. In detail, we investigated the wavelength dependence of the catalytic oxidation capabilities of a polymeric Eosin Y photocatalyst (**P1**) as well as a small molecule representative of the polymer bound Eosin Y moieties (**EY**) for the ambient oxygen mediated oxidation of triphenylphosphine. The highest catalyst efficiency was observed upon irradiation at 540 nm, the wavelength of the extinction maximum. Introduction of zinc(II) ions into the system proved to drastically influence the optical absorption properties of both catalysts which were rationalized by TDDFT calculations. The changes in the optical absorption properties were accompanied by pronounced influences on the wavelength‐dependent reactivity profiles, shifting the peak catalyst efficiency to shorter wavelengths and higher conversions, which are not predictable from the absorption spectra alone, highlighting the need for detailed photochemical action plot analyses. For **P1**, these changes were reversed by metal‐mediated SCNP formation upon base addition, giving access to a stimuli‐responsive polymeric photocatalyst. Detailed analysis of the photocatalytic action plots enabled the identification of 440 nm as a suitable wavelength for the realization of an on‐off switchable polymeric photocatalyst system.^[^ ^48^ ^]^ Our work is expected to serve as inspiration for the further exploration of the irradiation wavelength dependence of other photocatalytic transformations, eventually enabling a fundamentally enhanced understanding of photocatalytic reactions.

## Supporting Information

The authors have cited additional references within the Supporting Information.^[^ ^49^, ^50^, ^51^, ^52^, ^53^, ^54^, ^55^, ^56^, ^57^, ^58^, ^59^, ^60^, ^61^, ^62^, ^63^, ^64^, ^65^, ^66^, ^67^ ^]^ Data for this paper are available at radar4chem [https://radar.products.fiz‐karlsruhe.de/] at https://doi.org/10.22000/uju3k58znj7ahhm7.

## Author Contributions

S.G. (Conceptualization, Methodology, Formal analysis, Investigation, Writing — Original Draft, Review & Editing, Visualization, Project administration), A.K.F. (Investigation (Supporting), Writing — Review & Editing), J.O.H. (Writing — Review & Editing, Supervision), H.F. (Conceptualization, Writing — Review & Editing, Supervision), F.W. (Writing — Review & Editing, Supervision (Quantum Chemistry), C.B.‐K. (Conceptualization, Writing — Review & Editing, Supervision, Project administration, Funding acquisition), P.W.R. (Conceptualization, Writing — Review & Editing, Supervision, Project administration, Funding acquisition).

## Conflict of Interests

The authors declare no conflict of interest.

## Supporting information



Supporting Information

## Data Availability

The data that support the findings of this study are available in the supplementary material of this article.
